# Iterative Reconstruction Improves Both Objective and Subjective Image Quality in Acute Stroke CTP

**DOI:** 10.1371/journal.pone.0150103

**Published:** 2016-03-01

**Authors:** Fabian Flottmann, Jan Kabath, Till Illies, Tanja Schneider, Jan-Hendrik Buhk, Jens Fiehler, André Kemmling

**Affiliations:** 1 Department of Diagnostic and Interventional Neuroradiology, University Medical Center Hamburg-Eppendorf, Hamburg, Germany; 2 Institute of Neuroradiology, University Medical Center Schleswig Holstein, Lübeck, Germany; University Hospital-Eppendorf, GERMANY

## Abstract

**Purpose:**

Computed tomography perfusion (CTP) imaging in acute ischemic stroke (AIS) suffers from measurement errors due to image noise. The purpose of this study was to investigate if iterative reconstruction (IR) algorithms can be used to improve the diagnostic value of standard-dose CTP in AIS.

**Methods:**

Twenty-three patients with AIS underwent CTP with standardized protocol and dose. Raw data were reconstructed with filtered back projection (FBP) and IR with intensity levels 3, 4, 5. Image quality was objectively (quantitative perfusion values, signal-to-noise ratio (SNR), contrast-to-noise ratio (CNR)) and subjectively (overall image quality) assessed. Ischemic core and perfusion mismatch were visually rated. Discriminative power for tissue outcome prediction was determined by the area under the receiver operating characteristic curve (AUC) resulting from the overlap between follow-up infarct lesions and stepwise thresholded CTP maps.

**Results:**

With increasing levels of IR, objective image quality (SNR and CNR in white matter and gray matter, elimination of error voxels) and subjective image quality improved. Using IR, mean transit time (MTT) was higher in ischemic lesions, while there was no significant change of cerebral blood volume (CBV) and cerebral blood flow (CBF). Visual assessments of perfusion mismatch changed in 4 patients, while the ischemic core remained constant in all cases. Discriminative power for infarct prediction as represented by AUC was not significantly changed in CBV, but increased in CBF and MTT (mean (95% CI)): 0.72 (0.67–0.76) vs. 0.74 (0.70–0.78) and 0.65 (0.62–0.67) vs 0.67 (0.64–0.70).

**Conclusion:**

In acute stroke patients, IR improves objective and subjective image quality when applied to standard-dose CTP. This adds to the overall confidence of CTP in acute stroke triage.

## Introduction

In acute stroke triage, computed tomography perfusion (CTP) imaging provides clinically relevant information about the infarct core and tissue at risk for treatment decisions [1]. However, for detection of infarct core, CTP suffers from large measurement errors due to image noise compared to diffusion weighted magnetic resonance imaging [2]. Furthermore different perfusion processing algorithms and vendor specific software are known to produce different perfusion values with variable sensitivity and specificity to detect ischemia and infarct [3].

IR optimizes imaging data and thus reduces noise and artefacts, which leads to improved image quality in similar-dose protocols compared to traditional reconstruction methods such as filtered back projection (FBP), either permitting the usage of lower radiation doses or maintaining consistent image quality [4]. Combined with IR algorithms, the effectiveness of low-dose CTP was demonstrated in a clinical setting for a majority of acute stroke patients [5]. Nevertheless, it has not been examined whether IR leads to better identification of ischemic tissue in standard-dose CTP, and how IR performs across different CTP processing algorithms to predict infarct.

The purpose of our study was to examine (1) if IR, compared to standard FBP, improves CTP image quality in acute stroke patients, (2) how IR affects clinically relevant imaging features (i.e. ischemic core and perfusion mismatch), (3) how IR affects the predictive power for final infarct in CTP parameter maps (cerebral blood volume (CBV), cerebral blood flow (CBF), and mean transit time (MTT)).

## Materials & Methods

The study was performed with ethical review board approval (Ethik-Kommission der Ärztekammer Hamburg) and followed the Helsinki guidelines for Human experiments. The requirement of written informed consent was waived, only data from a retrospective anonymized database was used in this study. For retrospective data acquisition, inclusion criteria were a priori defined and applied consecutively to our database in a prospective chronological fashion. Inclusion criteria were as follows: 1) patients who received a multimodal computed tomography (CT) imaging protocol including a non-enhanced brain CT and CTP for the diagnosis of acute ischemic stroke between September 2012 and November 2013. 2) no prior major stroke. 3) visible perfusion deficit. 4) visible infarct lesion on follow-up imaging.

### Image acquisition and reconstruction

All exams were performed according to the department’s standard protocol on an iCT 256^™^ scanner (Philips Healthcare, Best, The Netherlands).

CT, CT angiography (CTA) and dynamic time resolved perfusion CT were performed in equal order. For native CT, the following scan parameters were used: collimation 64 x 0.625, pitch 0.297, rotation time 0.4 s, FOV 270 mm, tube voltage 120 kV, tube current 300 mA, 4.0 mm slice reconstruction. CTA: collimation 64 x 0.625, pitch 0.985, rotation time 0.4 s, FOV 220 mm, tube voltage 120 kV, 300 mAs, 2.0 mm slice reconstruction, 5 mm MIP reconstruction with 1 mm increment. CTP: collimation 64 x 1.25, rotation time 0.5 s, FOV 220 mm, tube current 80 kV, tube current 140 mAs, 5 mm slice reconstruction, slice sampling rate 1.8 s, scan time 72 s, biphasic injection with 40 ml of highly iodinated contrast medium with 400 mM/ml injected with 6 ml/s followed by 40 ml NaCl chaser bolus. All perfusion datasets were inspected for quality and excluded in case of severe motion artefacts. All CT exams were performed during clinical routine and therefore initially reconstructed with FBP according to the department’s standard parameters. Raw image data for study evaluation were transferred to a prototype processing software featuring iterative reconstruction with iDose4^™^ (Philips Healthcare, Best, The Netherlands). Image reconstructions were generated with IR using iDose level 3, 4 and 5, with theoretic noise reduction of 23%, 29% and 37%, respectively [6].

### CTP data post processing

Following either FBP or IR, perfusion raw data were processed for noise removal (motion correction and temporal smoothing) prior to calculation of parameter maps as implemented in dedicated perfusion software (VPCT-Neuro, Siemens Healthcare, Forchheim, Germany). Motion correction and tissue segmentation (skull stripping) was applied. Low band temporal noise removal was then performed to smooth the bolus attenuation curve for each voxel. First, each perfusion image at each time point was split into a spatially high and low band image after Fourier transformation with thresholds depending on the maximum and minimum frequencies of the original image. The temporal noise removal was then based on a gamma fit of the bolus curve per voxel using the low band part of each image at each time point (i.e. the part that contains most of the morphological information of the stroke lesion and large vessels), while the high band part of the image, containing most of the noise and high detail information such as small vessels and borders between cerebrospinal fluid (CSF) and brain parenchyma, was temporally averaged over the entire acquisition and added back to each low band smoothed image per time point as an off-set. This processed raw perfusion data bolus signal was then used to calculate perfusion parameter maps.

Raw perfusion CTP data were processed using four different post processing algorithms. Least mean square deconvolution (LMSD) and maximum slope (MS) based perfusion parameter maps [7] were calculated on a dedicated workstation for perfusion analysis (Syngo mmwp VE52A; Siemens Healthcare, Forchheim, Germany). Singular value decomposition deconvolution (sSVD) and block-circulant SVD (bSVD) perfusion parameter maps were calculated directly from the residue function using the Perfusion Mismatch Analyzer (PMA) Software (PMA, ASIST Group, Japan).

Non-parenchymal vascular voxels were automatically excluded by temporal intensity thresholding. Arterial voxels were identified automatically by identifying the vascular voxels with the earliest peak enhancement. The arterial and venous reference voxels were selected automatically under supervision. Perfusion datasets with incomplete or irregular arterial and venous attenuation time curves or incomplete ischemic territory coverage were excluded. All perfusion maps were converted to 5 mm slice thickness using nearest neighbor interpolation, thus image perfusion values remained unaltered.

### Objective image quality analysis

Objective image quality analysis was performed for IR (level 3, 4, 5) and FBP based CTP maps based on the LMSD algorithm. Three identical circular regions of interest (ROI) of 100 mm^2^ were placed with identical position in all calculated CTP parameter maps: (i) white matter (WM) and (ii) grey matter (GM) of the non-ischemic contralateral hemisphere within the middle cerebral artery (MCA) territory, and (iii) in the ischemic lesion as defined by CBV reduction and MTT increase. Quantitative measurements of perfusion parameters (CBV, CBF, MTT) and noise (standard deviation) were determined in each ROI. Signal-to-noise ratio (SNR) and contrast-to-noise ratio (CNR) were calculated for the CBV parameter. The SNR was defined as the mean CBV value divided by CBV image noise in each ROI. The CNR was defined as the difference in mean CBV values between ROI in GM and WM divided by the square root of the sum of their variance [5].

Signal quality of temporal parameter maps (MTT) was evaluated with respect to the number of error voxels. A problem with CTP algorithms is that brain voxel with very low cerebral blood volume and undetectable attenuation levels of bolus arrival cannot be assigned a discrete time value exhibiting a theoretical indefinite delay [8]. In MTT maps, brain voxels with very low cerebral blood volume and attenuation levels of bolus arrival below the limit of detection are marked as error voxels by the LMSD algorithm. The total number of error voxels was counted in IR and FBP based CTP maps and compared. Furthermore, the mean MTT was assessed in voxels presenting as error voxels when using FBP but not when using IR.

### Subjective image quality analysis and clinical rating

Subjective image quality analysis was performed for IR (level 3, 4, 5) and FBP based CTP maps based on the LMSD algorithm. One certified neuroradiologist (> 10 years experience) and two less experienced radiologists (< 2 years experience) performed scoring of the image data. The raters ranked (i) overall subjective image quality (1 = worst, 4 = best) according to level of image noise, visibility of ischemic lesion and negative effect of error voxels (ii) the presence of clinically significant ischemic core as a cut off for treatment decisions (as defined by visible CBV lesion) covering more than 1/3 of the MCA territory [9] and (iii) the presence of a clinically relevant perfusion mismatch as defined by a demarcated MTT lesion covering approximately 20% more MCA territory than the CBV lesion [10]. Image viewing was performed on a PACS-Workstation (PACS-IW, GE Healthcare, Milwaukee, MI). Images were displayed anonymously in random order and raters were allowed to scroll through the complete dataset. Infarct core in CBV maps and tissue at risk of infarction in CBF and MTT maps was evaluated using a fixed window-level at the optimal threshold for infarct with variable window width for optimal contrast [7].

### Predictive power for final infarct of IR and FBP based CTP maps

Receiver operating characteristic (ROC) curve analysis was performed to evaluate the predictive power to detect final infarct in CTP maps based on different commonly employed perfusion algorithms (LMSD, MS, bSVD, sSVD). This was done for IR and FBP based CTP maps, respectively (supplemental material).

Quantitative perfusion maps were calculated for CBV, CBF and MTT. Infarct lesions on follow-up imaging CT were segmented manually. Final tissue outcome was classified voxel-wise as a binary response variable (1 = infarct, 0 = no infarct). All follow-up images were then registered to the baseline time average image of the CTP dataset (Analyze 11.0, AnalyzeDirect). The perfusion parameter maps and the follow up infarct map of each patient were converted to a voxel-wise data matrix (Matlab R2014a). The data sample space of each patient consisted of all brain parenchymal voxels covered by CTP (both hemispheres excluding vessels and CSF). The voxel-wise data matrix was exported to R (Version 3.02, EPI package) for ROC curve analysis with stepwise per unit increasing CTP parameter thresholds. Thus, predictive power for tissue outcome was determined by the area under the ROC curve (AUC) resulting from the overlap of stepwise thresholded CTP maps with follow-up infarct lesions.

### Statistical analysis

Perfusion values and noise levels (CBV, CBF, MTT), SNR and CNR (CBV), as well as the number of error voxels (MTT) and differences of AUC between FBP and IR were compared with the analysis of variance (ANOVA) for repeated measures. Subjective image quality was tested using the Wilcoxon test for repeated measures. Changes in assessment of perfusion mismatch were compared using the McNemar’s test on paired nominal data.

The level of significance was defined as P ≤ 0.05. P values are displayed Bonferroni-corrected.

## Results

### Study group characteristics

Six of the 32 patients who met the inclusion criteria were excluded because of movement artifacts. In addition, one patient was excluded due to a program error of the PMA software. One patient was excluded due to absence of a perfusion deficit and one patient due to absence of an ischemic lesion in follow up imaging.

The remaining 23 patients (22% (5/23) male, age = 73±18 years) were included in the study group (Table 1). All patients showed symptoms of acute ischemic stroke (mean National Institutes of Health Stroke Scale (NIHSS) score 12) and demonstrated a perfusion deficit in CTP. 83% of patients showed a vessel occlusion in cerebral circulation on the baseline CTA. Intravenous (IV) recombinant tissue plasminogen activator (rtPA) was given in 43%, 13% received additional mechanical therapy, and 22% received mechanical therapy only.

**Table 1:** Baseline characteristics and therapy of 23 patients.

Baseline characteristics	All Patients
Subjects, n	23
Age, years, mean (SD)	73.5 (18.0)
Male sex, n (%)	5 (22)
Admission NIHSS, median (IQR)	12 (9)
Vessel occlusion	
Internal carotid artery (ICA), n (%)	2 (8.6)
Carotid-T, n (%)	2 (8.6)
Middle cerebral artery (MCA) main stem, n (%)	11 (47.8)
Posterior cerebral artery (PCA), n (%)	4 (17.4)
Other, n (%)	4 (17.4)
Therapy	
IV rtPA, n (%)	8 (34.8)
IV rtPA and mechanical, n (%)	3 (13.0)
Mechanical only, n (%)	5 (21.7)
No therapy, n (%)	7 (30.4)

### Objective image quality

#### Mean perfusion parameter values

Table 2 shows the average values of CBV, CBF and MTT in GM, WM and the ischemic lesion for the four different sets of CTP reconstructions. There were no significant differences in CBV and CBF between FBP and IR. Furthermore, there were no significant differences in MTT in GM and WM. However, in the ischemic lesion, MTT values were significantly higher in IR levels 3, 4 and 5 (p = 0.006; 0.002; 0.002 respectively) compared to FBP.

**Table 2:** Mean perfusion parameter values.

	Mean (95% confidence interval)				
IR level	FBP	IR level 3	IR level 4	IR level 5	*P* value*
CBV (ml/100 g)					
Gray matter	3.7 (3.4–4.0)	3.5 (3.2–3.8)	3.6 (3.3–3.9)	3.9 (3.6–4.2)	0.727
White matter	1.8 (1.6–1.9)	1.7 (1.6–1.9)	1.7 (1.6–1.9)	1.8 (1.7–2.0)	0.837
Ischemic lesion	2.5 (2.1–2.8)	2.3 (2.0–2.7)	2.4 (2.0–2.7)	2.6 (2.2–2.9)	1.000
CBF (ml/100 g/min)					
Gray matter	68.9 (62.8–74.9)	65.0 (59.7–70.4)	66.9 (61.3–72.4)	73.4 (66.9–80.0)	0.458
White matter	32.5 (30.0–35.0)	30.6 (28–33.3)	30.8 (28.1–33.5)	32.6 (29.8–35.5)	1.000
Ischemic lesion	25.7 (19.4–31.9)	22.6 (16.4–28.7)	21.8 (15.8–27.7)	23.4 (15.8–30.9)	1.000
MTT (s)					
Gray matter	3.6 (3.2–3.7)	3.5 (3.3–3.7)	3.4 (3.2–3.6)	3.4 (3.2–3.5)	1.000
White matter	3.6 (3.4–3.8)	3.7 (3.5–4.0)	3.8 (3.6–4.1)	3.9 (3.6–4.2)	0.101
Ischemic lesion	9.4 (8.0–10.7)	10.1 (8.7–11.5)	10.6 (9.2–12.0)	10.6 (9.1–12.0)	0.002

CBV, CBF and MTT values for FBP vs. IR with 3 different levels of intensity.

* p-value for comparison of FBP vs IR level 5.

MTT values between GM and WM did not differ using FBP and IR level 3 (p = 0.46 and 0.075, respectively). However, in IR levels 4 and 5, MTT values were significantly different between GM and WM (p = 0.017 and p = 0.002, respectively).

#### Noise, SNR and CNR

Table 3 displays the measurements of noise for CBV, CBF and MTT in GM, WM and the ischemic lesion, the signal-to-noise ratio for CBV in GM, WM and the ischemic lesion as well as contrast-to-noise ratio for CBV. There were significant differences of noise levels for CBV in WM and for CBF in the ischemic lesion between FBP and IR level 5.

**Table 3:** Noise, signal-to-noise ratio and contrast-to-noise ratio.

	Mean (95% confidence interval)				
IR level	FBP	IR level 3	IR level 4	IR level 5	*P* value
Noise (SD)					
CBV					
Gray matter	0.6 (0.5–0.7)	0.6 (0.5–0.7)	0.6 (0.5–0.7)	0.6 (0.5–0.7)	0.543
White matter	0.5 (0.4–0.6)	0.4 (0.4–0.5)	0.4 (0.4–0.5)	0.4 (0.4–0.5)	0.027
Ischemic lesion	0.8 (0.7–1.0)	0.7 (0.6–0.9)	0.8 (0.6–0.9)	0.8 (0.7–0.9)	0.216
CBF					
Gray matter	14.3 (12.4–16.1)	13.6 (11.6–15.7)	14.0 (12.2–15.8)	14.0 (12.0–16.9)	1.000
White matter	11.1 (9.6–12.7)	9.7 (8.5–10.9)	10.0 (8.9–11.1)	10.3 (9.3–11.4)	1.000
Ischemic lesion	13.2 (11.0–15.4)	10.7 (9.2–12.3)	10.4 (9.0–11.8)	10.8 (8.9–12.6)	0.022
MTT					
Gray matter	0.9 (0.4–1.4)	0.9 (0.5–1.2)	0.8 (0.5–1.2)	0.7 (0.4–1.0)	1.000
White matter	5.9 (5.0–6.9)	6.3 (4.9–7.7)	6.4 (4.9–7.8)	5.8 (4.8–6.8)	1.000
Ischemic lesion	1.3 (0.9–1.6)	1.4 (1.1–1.8)	1.6 (1.2–1.9)	1.5 (1.1–1.8)	0.267
Signal-to-noise ratio (CBV)					
Gray matter	6.4 (5.7–7.1)	6.8 (5.9–7.6)	6.7 (6.0–7.6)	7.2 (6.4–8.0)	0.026
White matter	3.8 (3.1–4.5)	4.3 (3.6–5.0)	4.4 (3.7–5.2)	4.6 (3.9–5.3)	< 0.001
Ischemic lesion	3.1 (2.6–3.7)	3.5 (2.7–4.3)	3.4 (2.8–4.1)	3.5 (2.8–4.3)	0.069
Contrast-to-noise ratio (CBV)					
GM vs WM	2.4 (2.1–2.8)	2.6 (2.2–3.0)	2.6 (2.2–3.0)	2.8 (2.4–3.2)	0.003

Noise and signal-to-noise ratio of gray matter, white matter and ischemic lesion and contrast-to-noise ratio for FBP vs. IR with 3 different levels of intensity.

* p value for comparison of FBP vs IR level 5

The signal-to-noise ratio was significantly higher in IR 5 vs. FBP for CBV in GM (p = 0.026). In WM, SNR was significantly higher in IR levels 3, 4 and 5 (p = 0.010; 0.008; 0.0004 respectively) compared to FBP. In the ischemic lesion, the mean SNR was higher in IR 5 vs. FBP, but not statistically significant (p = 0.069). The contrast-to-noise ratio was significantly higher in IR level 5 vs. FBP (p = 0.003).

#### Lower number of error voxels in MTT maps

**Fig 1:**
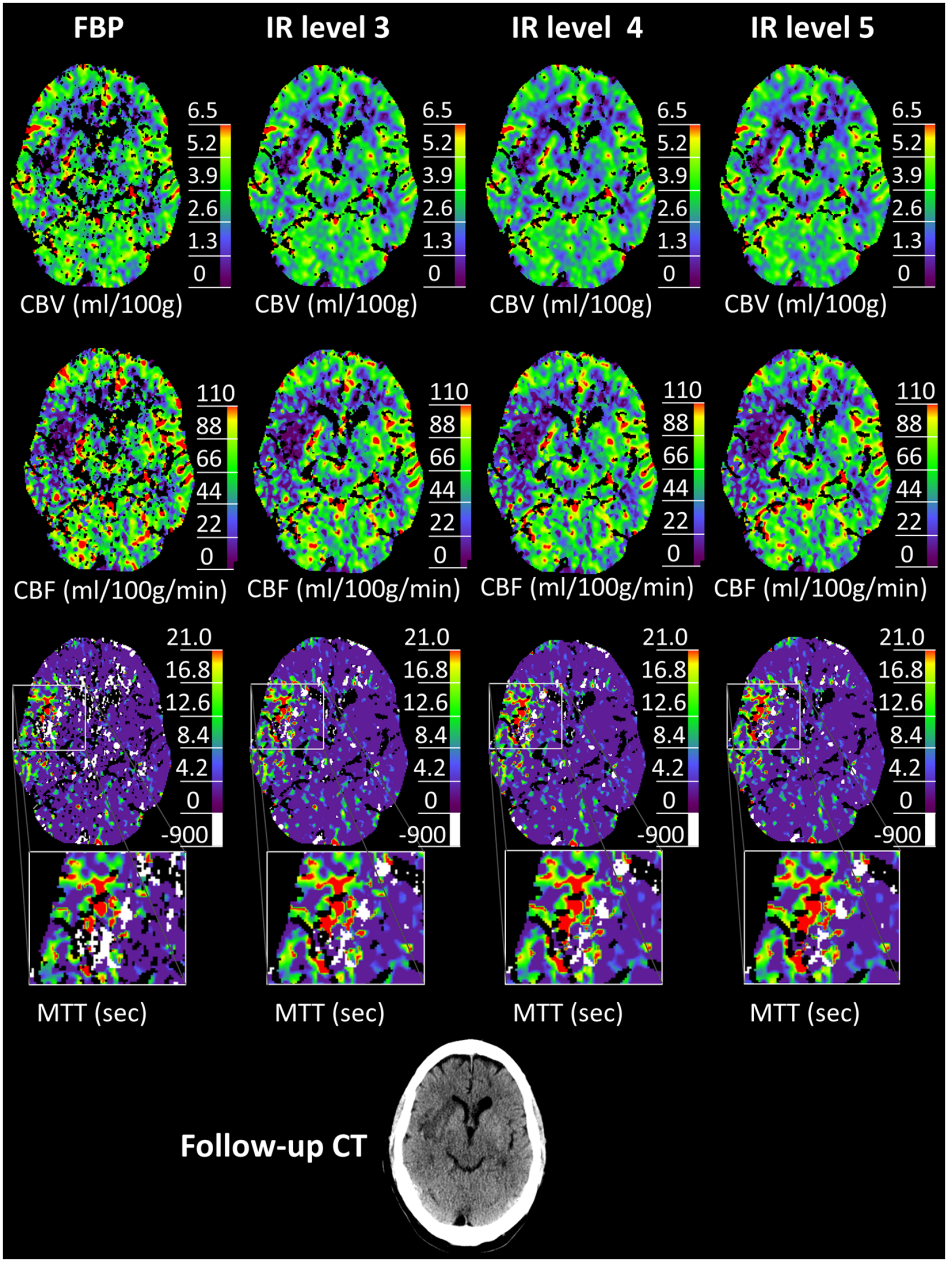
Example CTP images 1. Note the improved image quality from FBP to IR levels 3–5 (rated 1.3, 2.0, 3.6 and 3.0, respectively). With rising levels of IR, error voxels (i.e. voxels without perfusion information, white) diminished in favor of voxels with perfusion information (colored). For this patient, as for 19 out of 23 patients, the assessment of a relevant perfusion mismatch did not depend on the reconstruction algorithm.

**Fig 2:**
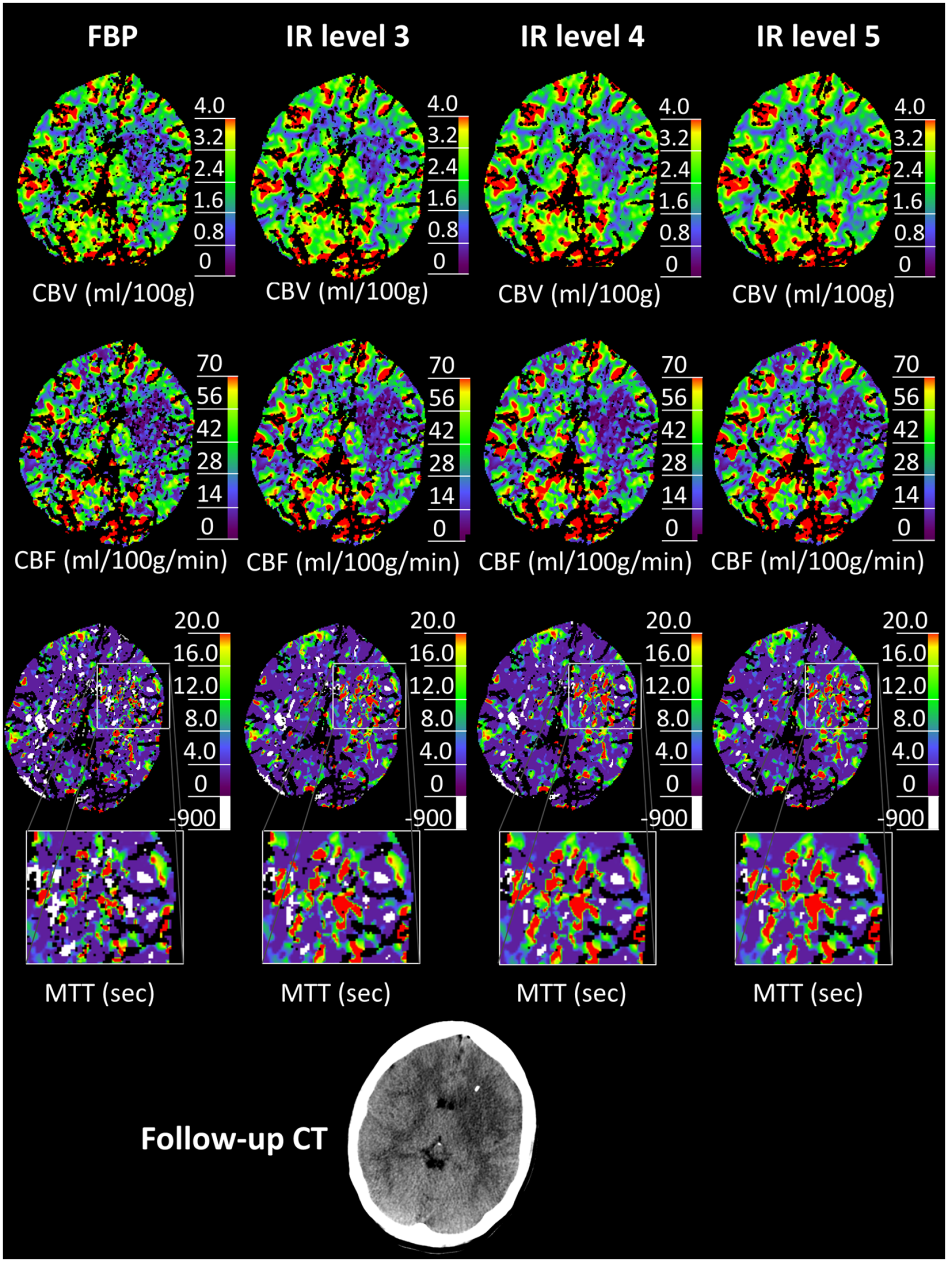
Example CTP images 2. In this case, the clinical decision differed from FBP (no relevant perfusion mismatch) to IR level 3–5 (visible relevant perfusion mismatch). While there was no change of the ischemic core visible in CBV, accentuated ischemic lesions became visible in CBF and MTT with increasing IR levels. In 4 of 23 cases, there was a change in the assessment of a perfusion mismatch.

Error voxels in MTT maps were designated with a value of -900 by the LMSD algorithm in brain areas with attenuation levels of bolus arrival below limit of detection. Figs 1 and 2 show example CTP images in which the error voxels are colored white (MTT map only).

**Fig 3:**
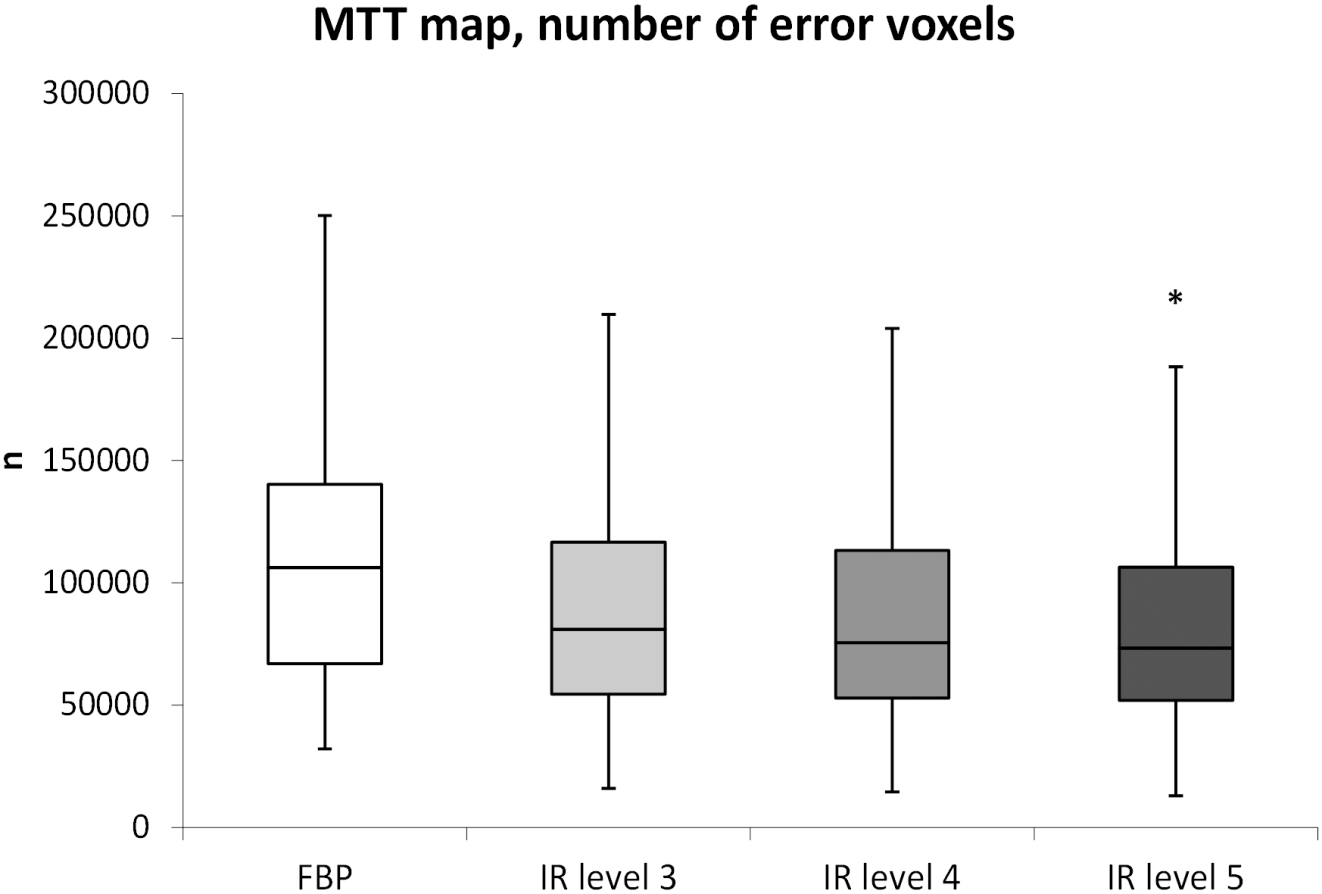
MTT map, number of error voxels. The amount of error voxels (i.e. voxels without perfusion information) was significantly reduced between FBP and IR level 5. (* p = 0.004).

The mean number of error voxels detected in MTT maps based on FBP was 108099 ± 26147 (Fig 3B). The number of error voxels decreased with increasing IR level: 92371 ± 24488 for level 3, 88636 ± 24853 for level 4 and 81317 ± 19710 for level 5. The mean number of error voxels in IR 5 was reduced by 24.8% compared to FBP, and this difference (26782 [7257.3–46306.8]) was statistically significant (p = 0.004).

The mean MTT value in the ischemic lesion was 9.4 using FBP. Voxels in the ischemic lesion that were classified as error voxels using FBP and converted to voxels with perfusion information using IR had mean MTT values of 5.2 in IR level 3, 5.3 in IR level 4 and 4.9 in IR level 5.

### Subjective image quality

Subjective image quality of CBV, CBF and MTT maps was assessed independently by 3 different raters. Quality criteria were image noise, amount of error voxels and visibility of ischemic lesion in comparison to healthy tissue.

**Fig 4:**
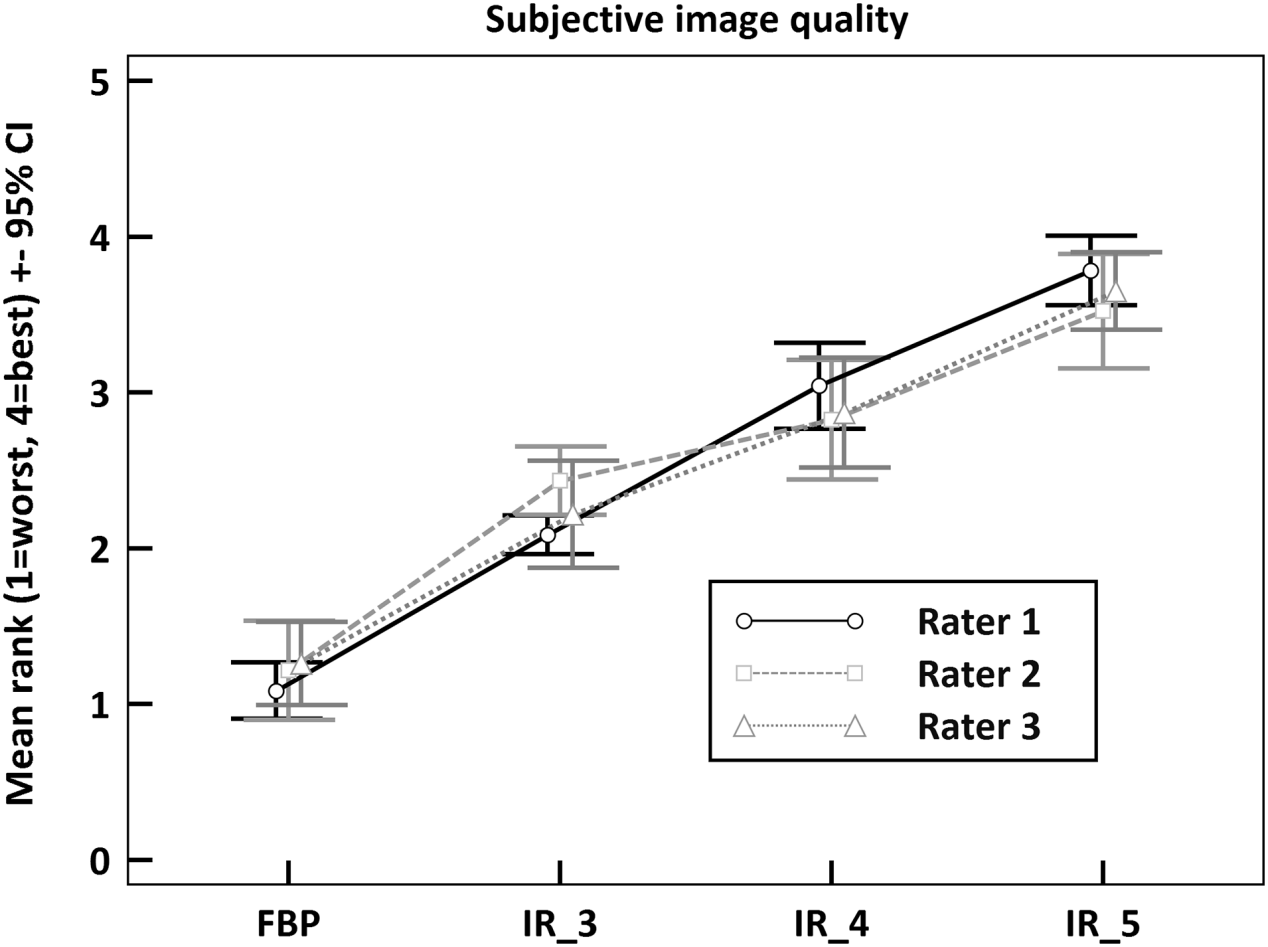
Subjective image quality. Overall subjective image quality of CTP maps ranked by 3 raters (1 = worst, 4 = best). CTP images for each patient acquired with FBP and IR levels 3–5 were rated independently by 3 raters in terms of overall image quality (image noise, amount of error voxels, visibility of ischemic lesion). 95% CI are displayed for the mean rank assigned by a rater to the respective reconstruction method (FBP vs. IR levels 3–5.) All IR levels were ranked significantly higher than FBP (p<0.05).

Among all raters, there was a strong correlation between IR level and image quality. FBP was ranked lowest, whereas IR level 5 was ranked highest in terms of image quality (Fig 4). The difference between FBP and all IR levels was statistically significant for all raters (p<0.05), as was the difference between IR level 3 and IR level 5. However, the difference between IR level 3 and 4 was not significant for 2 of the 3 raters.

### Clinical decision making

15 of 23 patients had an infarction in the territory of MCA. These patients were visually assessed for clinically significant ischemic core (defined as visually demarcated CBV lesion above 1/3 of media territory). Core lesion in 5 patients was rated as significant with no different ratings between FBP and IR.

**Fig 5:**
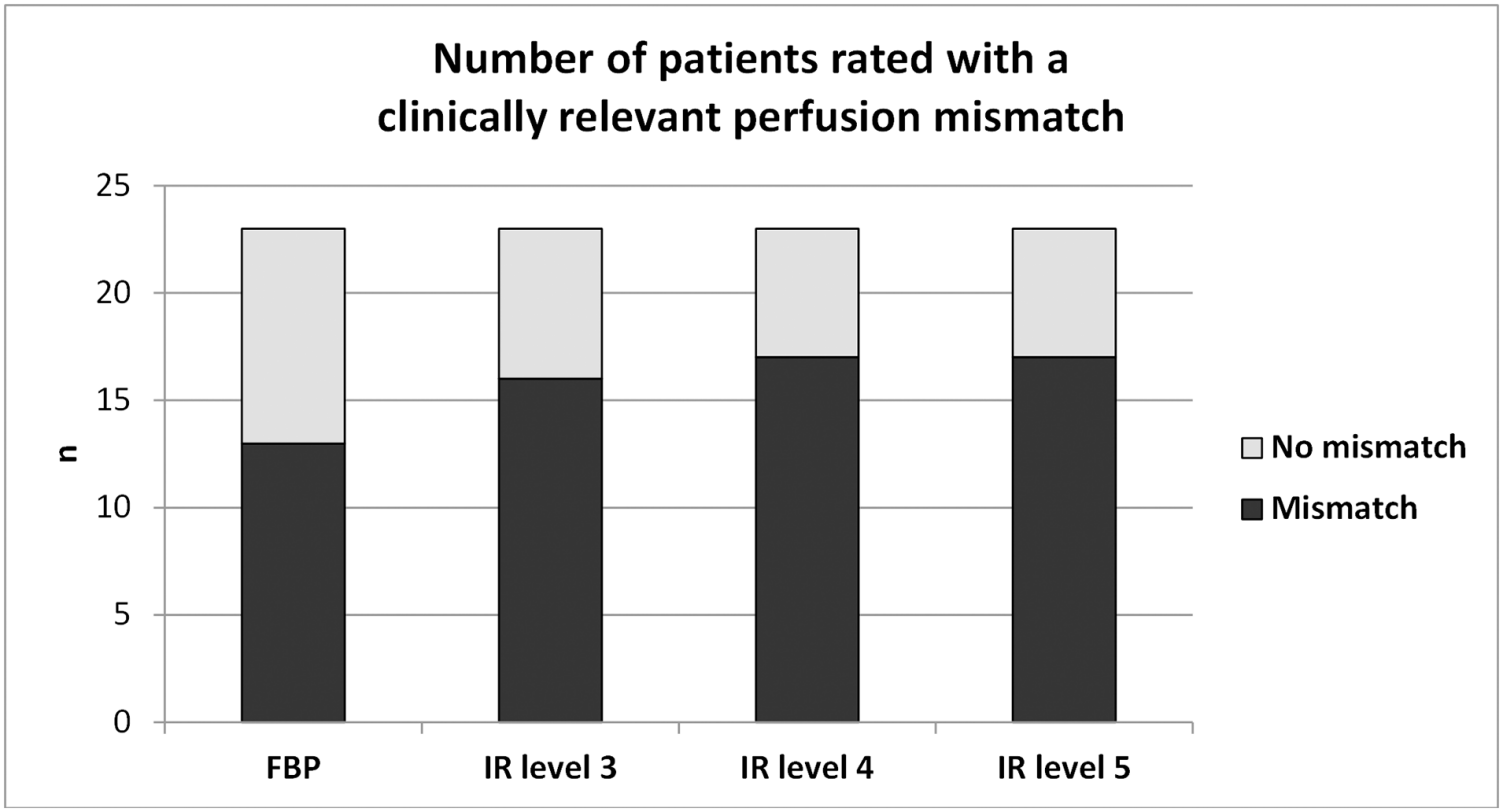
Number of patients rated with a clinically relevant perfusion mismatch. Number of patients rated with a clinically relevant perfusion mismatch (> 20% mismatch between CBV and MTT lesion). Using FBP, 13 patients were rated positively. This number increased to 17 using IR level 5. The difference was not statistically significant.

Fig 5 shows the number of patients rated with a clinically relevant perfusion mismatch as visually assessed by 3 raters (> 20% mismatch between CBV and MTT lesion). For FBP, 13 of 23 patients were rated positively for a mismatch. In 4 patients, there was a change in the assessment of a clinically relevant perfusion mismatch using IR (example case shown in Fig 2). The number of patients with mismatch rose from 13 in FBP to 17 in IR level 5. The difference was not statistically significant (p = 0.25, FBP vs IR 3, p = 0.125, FBP vs. IR 4 and 5).

### Predictive power and correlation with final infarct lesion

**Fig 6:**
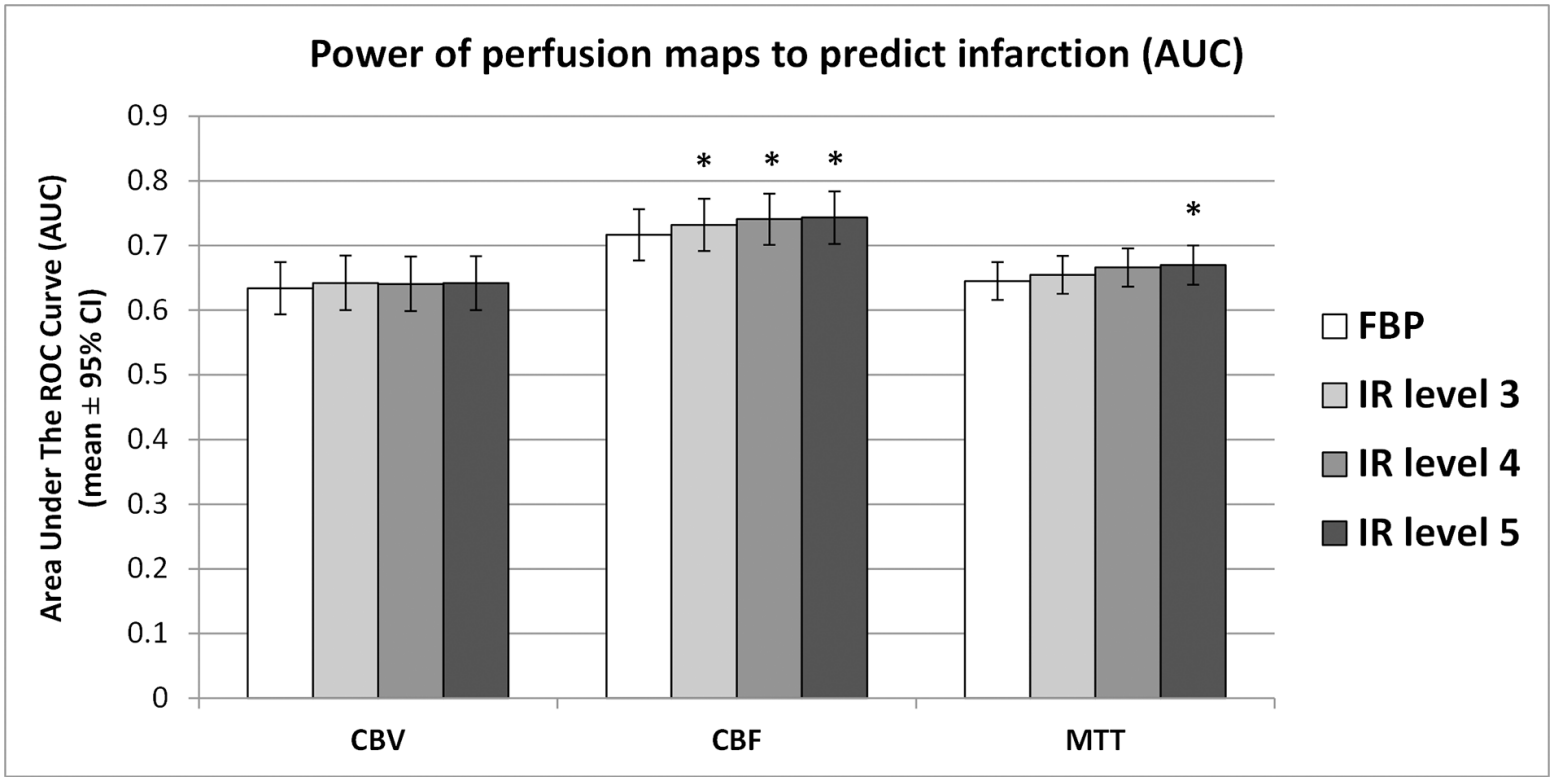
Power of perfusion maps to predict infarction represented by AUC. Predictive power for CBV, CBF and MTT map across three different IR levels and FPB. CBV showed no significant changes. CBF and MTT predictive power changed statistically significant between FBP and IR, with relative differences < 4%. (* = p<0.05).

ROC curve analysis was performed to evaluate the power of final infarct prediction (AUC) in FBP and IR based CTP maps. Fig 6 shows the predictive power using FBP reconstruction vs. three different IR levels of CBV, CBF and MTT maps.

The CBV map had mean AUC values between 0.63 and 0.64 to predict final infarct from thresholding. The relative difference between FBP and IR level 5 was 1.2%. There were no significant differences between FBP and IR.

The CBF map had mean AUC values between 0.72 and 0.74. The relative AUC difference between FBP and IR level 5 was 3.7%. The AUC of FBP was significantly lower than IR level 3, 4 and 5 (p<0.05).

The MTT map had mean AUC levels between 0.65 and 0.67. The relative difference between FBP and IR level 5 was 3.8%. The AUC of FBP was significantly lower than IR level 5 (p<0.05).

All perfusion parameter maps were tested for 3 additional post-processing algorithms with similar results (S1 File).

## Discussion

This study examined the influence of IR on CTP image quality in acute stroke patients. IR significantly improved objective image quality: while SNR and CNR were improved, the amount of error voxels in MTT maps decreased. Accordingly, there was a strong correlation between IR level and subjective image quality. Interestingly, all perfusion parameter values remained unchanged, with the exception that MTT was increased in the ischemic lesion. Visually, IR influenced the perception of relevant perfusion mismatch (assessed by CBV and CBF/MTT lesion difference) which was more frequent in higher level IR than FBP. However, no patient presenting with a large ischemic core (> 1/3 of MCA territory) was rated differently using IR. Our systematic analysis of CBF, CBV and MTT maps and their respective predictive power (AUC) for infarction revealed minor relative improvements (< 4%) using IR compared to FBP.

The improvements in subjective and objective image quality using IR are in accordance with a previous study on IR in native head CT [11] as well as on IR added to half-dose CTP in AIS [5]. The consistency of perfusion parameter values using IR in GM, WM and ischemic lesions had been shown in low-dose CTP [12], however, they did not report a significant elevation of MTT levels in the ischemic lesion.

MTT values between GM and WM did not differ significantly using FBP. However, there were significant differences using IR, suggesting an improved discrimination of MTT values between GM and WM. These findings are consistent with recent results found with improved CT perfusion algorithms [13].

In MTT maps, the LMSD algorithm assigned values of -900 to all brain voxels in which the mean transit time could not be calculated (error voxels). When these voxels were reduced by IR, they were exchanged for voxels with perfusion information. As these converted voxels had low MTT values compared to all voxels inside the ischemic lesion, it is unlikely that the observed MTT elevation is caused by the reduction of error voxels. A reduction of error voxels could however lead to less image noise and a better demarcation of the ischemic lesion, which could explain the tendency towards the perception of a perfusion mismatch by the raters. Furthermore, as the number of error voxels decreased across IR levels, high IR level perfusion parameter maps contain more voxels with perfusion information, which is especially interesting for automated evaluation of CTP.

We found IR to increase MTT levels inside the ischemic lesion, and compared to FBP the prevalence of a relevant perfusion mismatch changed from "not present" to "present" in 4 out of 23 patients. Another study would be necessary to evaluate whether this finding is statistically significant when examined in a larger patient cohort. Our study did not take into account important covariates such as recanalization status and time interval until recanalization which would require a much larger study population. Furthermore, a combination of IR with advanced acquisition technologies, different post-processing algorithms and/or multi-parametric approaches including clinical data should be investigated, as it might even further improve the diagnostic quality of CTP in AIS.

In conclusion, IR in standard-dose CTP improved both objective and subjective image quality. Thus, it adds to the confidence in CTP for acute stroke triage.

## Supporting Information

S1 File Predictive power for CBV, CBF and MTT map using 4/3 algorithms in 3 different IR levels and FPB. A systematic analysis of the predictive power using FBP reconstruction vs. three different IR levels of CBV, CBF and MTT maps generated with 4 (3 for MTT) different post-processing algorithms.
(PDF)

S2 File Anonymized minimal data set. (XLSX)
