# Supplementary material for: Iterative Reconstruction Improves Both Objective and Subjective Image Quality in Acute Stroke CTP
Source: PLoS One. 2016 Mar 1;11(3):e0150103. doi: 10.1371/journal.pone.0150103 (PMC4773110; doi:10.1371/journal.pone.0150103)
Supplement: S1 File — A systematic analysis of the predictive power using FBP reconstruction vs. three different IR levels of CBV, CBF and MTT maps generated with 4 (3 for MTT) different post-processing algorithms. (PDF) [file pone.0150103.s001.pdf]

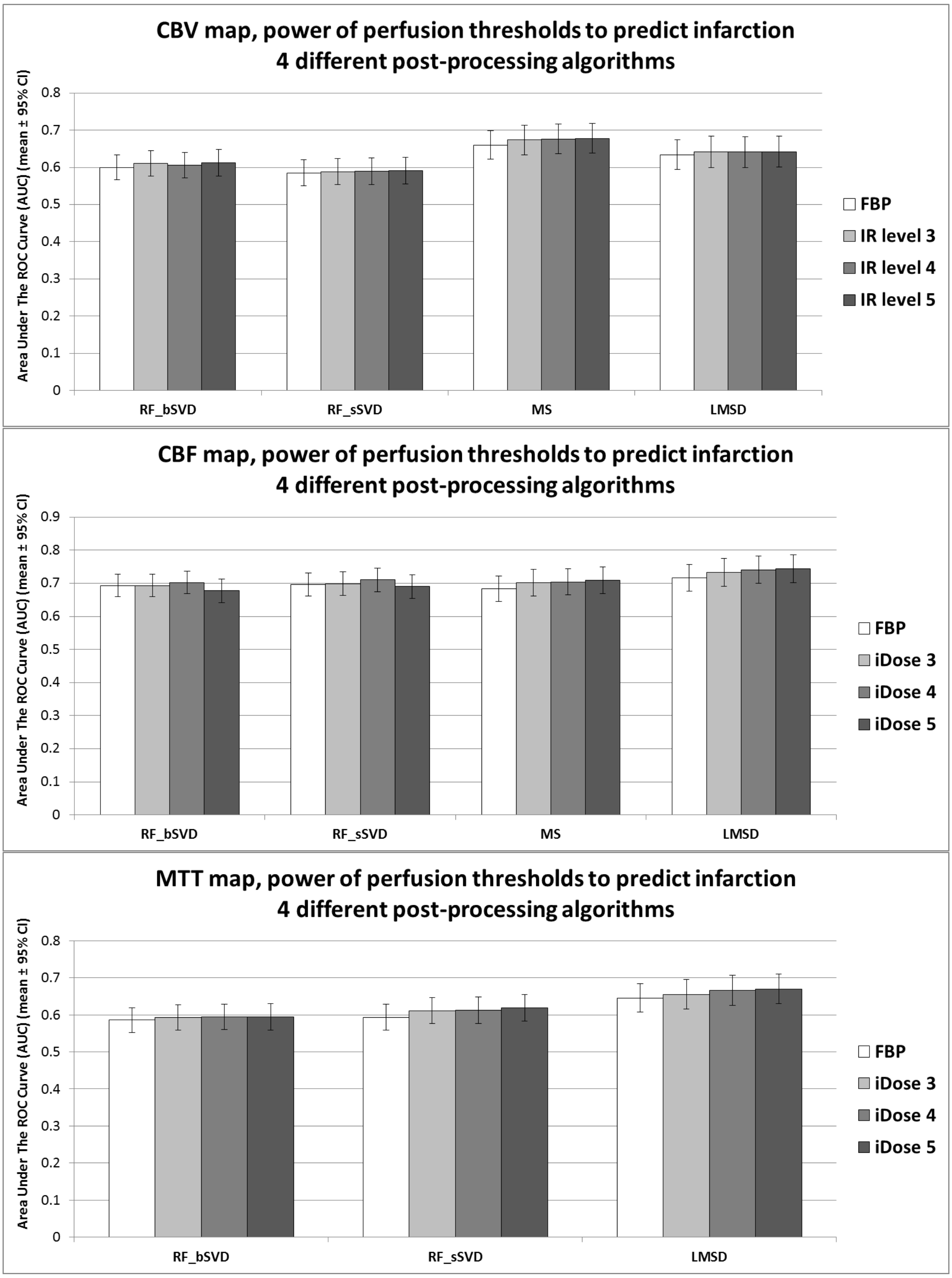

Predictive power for CBV, CBF and MTT map using 4/3 algorithms in 3 different IR levels and FPB. A systematic analysis of the predictive power using FBP reconstruction vs. three different IR levels of CBV, CBF and MTT maps generated with 4 (3 for MTT) different post-processing algorithms.

CTP from 23 acute stroke patients were reconstructed using FBP and three different IR levels (3, 4, 5). For each reconstruction method, CBV, CBF and MTT maps were generated using the following post-processing algorithms: Singular value decomposition deconvolution (sSVD) and block-circulant SVD (bSVD) using residue function (RF), as well as least mean square deconvolution (LMSD) and maximum slope (MS, not available for MTT map).

The CBV map showed mean AUC values between 0.59 (RF_sSVD using FBP) to 0.68 (MS using IR level 5). The biggest AUC difference between IR level 5 and FBP was 0.018 [0.003-0.0332] (p=0.0119) in the MS post-processing algorithm group (+2.8 % compared to FBP). In the CBF map, mean AUC levels lay between 0.68 (MS using FBP) and 0.74 (LMSD using IR Level 5), while the biggest difference between IR level 5 and FBP was 0.027 [0.008-0.0453] (p=0.002), in the LMSD post-processing algorithm group (+3.7% compared to FBP). The MTT map had AUC levels between 0.59 (RF_bSVD at FBP) and 0.67 (LMSD at IR level 5), while the highest difference between IR level 5 and FBP was 0.025 [0.001-0.049] (p=0.038) in the LMSD group (+3.8 % compared to FBP).

The maximum relative difference observed for any of the post-processing algorithms when comparing FBP to IR level 5 was 3.8%.
